# Taking a Closed-Book Examination: Decoupling KB-Based Inference by Virtual Hypothesis for Answering Real-World Questions

**DOI:** 10.1155/2021/6689740

**Published:** 2021-02-22

**Authors:** Xiao Zhang, Guorui Zhao

**Affiliations:** ^1^School of Mechanical Electronic and Information Engineering, China University of Mining and Technology, Beijing 100083, China; ^2^Coal Mining Research Institute, China Coal Technology and Engineering Group, Beijing 100013, China

## Abstract

Complex question answering in real world is a comprehensive and challenging task due to its demand for deeper question understanding and deeper inference. Information retrieval is a common solution and easy to implement, but it cannot answer questions which need long-distance dependencies across multiple documents. Knowledge base (KB) organizes information as a graph, and KB-based inference can employ logic formulas or knowledge embeddings to capture such long-distance semantic associations. However, KB-based inference has not been applied to real-world question answering well, because there are gaps among natural language, complex semantic structure, and appropriate hypothesis for inference. We propose decoupling KB-based inference by transforming a question into a high-level triplet in the KB, which makes it possible to apply KB-based inference methods to answer complex questions. In addition, we create a specialized question answering dataset only for inference, and our method is proved to be effective by conducting experiments on both AI2 Science Questions dataset and ours.

## 1. Introduction

Teaching machines to answer complex questions like human beings is a very challenging task at the intersection of nature language processing (NLP), information retrieval (IR), and artificial intelligence (AI), which mainly needs three techniques, i.e., question understanding, answer retrieval, and inference. There are three subtasks specifically to evaluate the corresponding techniques: Question Answering over Knowledge Base (KBQA) is a typical task to evaluate question understanding; Text Retrieval Question Answering (TREC QA) and Reading Comprehension (RC) are good tasks to evaluate answer retrieval and answer selection; Link Prediction and Knowledge Base Completion (KBC) are traditional tasks to evaluate inference.

After achieving progress in these subtasks, researchers begin to turn their passion to more comprehensive and complex question answering (QA) tasks. Allen Institute for Artificial Intelligence (AI2) proposes a science test which is a real-world examination for elementary students and middle school students, and it is even viewed as a standardized measure of AI. An example question in a science test is shown as follows. Q1: Peach trees have sweet-smelling blossoms and produce rich fruit. What is the main purpose of the flowers of a peach tree? (Answer is A.)

- To attract bees for pollination
- To create flower arrangements
- To protect the tree from disease
- To feed migratory birds

Such complex questions are hardly solved by any single technique mentioned above, and it is also difficult to effectively combine these techniques, which makes the task far from solved.

Retrieval can get effect instantly on a question whose answer (or some keywords inside) is near the question in background corpus, and the method achieved the best performance on 8th Grade Science Challenge [1]. However, those methods can do nothing to a question whose answer does not occur in the same document with the question, and they cannot capture long-distance dependencies across documents which furnish evidence for choosing the answer. This limitation makes retrieval have little space to improve with fixed corpus. According to [2], 77% of questions need inference, and retrieval is not viewed as true artificial intelligence [1].

**Figure 1:**
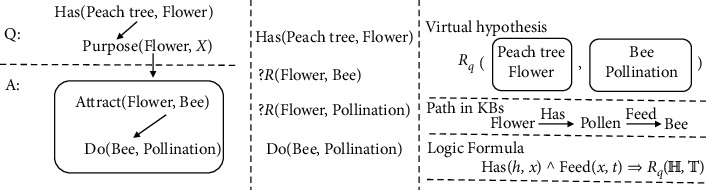
(a) A recursive structure obtained from semantic parsing. (b) Atomic hypotheses obtained by unfolding recursive structure. (c) A virtual hypothesis and its evidences.

On the other hand, knowledge bases (KBs) are graph-structured background data which contain vast long-distance semantic associations. Inference on KB is expected to capture such long-distance semantics as evidence by logic formulas [3, 4] and knowledge graph embedding [5, 6], and it has been proven to be effective in link prediction on KB and KBC task. However, KB-based inference does not fit real-world QA well; there are two reasons:

1. There is a gap between a natural language question and the semantic structure in KBs. Semantic parsing is used to transform a natural language to the semantic structure on the KB; e.g., for Q1, the question is expected to be Purpose (Flower, *X*), and its correct answer is expected to be Attract (Flower, Bee) ∧ Do (Be, Pollination). However, it is difficult to obtain such precise structures, because the quality of semantic parsing is far from satisfactory [7].
2. Even if the question can be precisely transformed into a structure by semantic parsing, complex structure in KBs is not appropriate as a hypothesis for inference. In inference, a hypothesis is a candidate proposition which needs to be proven by evidence; e.g., we substitute *X* in Purpose (Flower, *X*) with the candidate answer (as Figure 1(a) shows), and the recursive structure can be viewed as the hypothesis for inference. However, formulas used to infer such a complex structure should have a form like *R*1 (*X*, *Y*) ∧*R*2 (*Y*, *Z*) ∧ …⇒ Purpose (*X*, (Attract (*X*, *Y*) ∧ Do (*Y*, *Z*))), which is far from frequent in KBs and difficult to be found by formula learners.

An intuitive solution to complex hypothesis is to unfold the recursive structure and divide it into several atomic hypotheses; e.g., the structure in Figure 1(a) is unfolded into four triplets by establishing relationships between Flower and the two entities in the answer, shown in Figure 1(b), i.e., Has (Peach tree, Flower) ∧ ?*R* (Flower, Bee) ∧ ?*R* (Flower, Pollination) ∧ Do (Bee, Pollination). However, after unfolding and dividing, some structural information in the original question is missing, which embodies two aspects:

- There are no explicit relations between some entities. After unfolding, the original relation between the question and the answer (e.g., Purpose in Figure 1(a)) is no longer the relation of some atomic triplets. For example, in Figure 1(b), the relation of Flower, Pollination is unknown, and the relation of Flower, Bee is not Attract anymore because of Purpose's influence.
- There are no associations among atomic hypotheses. After dividing, we assume that atomic hypotheses are independent and each atomic hypothesis is inferred by its specific formulas. For example, after dividing, Eat (Bee, pollen) ⇒ Do (Bee, Pollination) which supports Do (Bee, Pollination) is irrelevant with Has (Peach tree, Flower). Actually, there should be associations among these hypotheses, and it is these associations that make atomic hypotheses the original question.

To resolve these problems, this paper proposes decoupling KB-based inference from question answering by transforming a complex QA pair into a virtual high-level hypothesis on the KB.

- For the entity pairs which have no explicit relations, we create a virtual relation Rq to replace unknown relations of entity pairs, and represent Rq into distributed semantic space according to the linguistic expressions of the question. We expect different dimensions of Rq can capture relations of different entity pairs. For example, Rq (Flower, Pollination) and Rq (Flower, Bee) focus on different aspects of Rq.
- For the atomic hypotheses among which there are no associations, we create a virtual hypothesis Rq (*H*, *T*) to combine all possible atomic hypotheses, and adapt original KB-based inference methods to infer it. For example, atomic hypotheses in Figure 1(b) are combined into Rq ({Peach Tree, Flower}, {Bee, Pollination}) (in Figure 1(c)). Therefore, the virtual hypothesis is treated as a whole and can be supported by evidences obtained from any pair of entity *h* ∈ *H* and entity *t* ∈ *T*. For example, in Figure 1(c), a path $\text{Flower}\overset{\text{Has}}{⟶}\text{Pollen}\overset{\text{Feed}}{⟶}\text{Bee}$ on the KB can produce a formula Has (*h*, *x*) ∧ Feed (*x*, *t*) ⇒ Rq (*H*, *T*), which is an evidence for the virtual hypothesis. At last, we build a joint inference model to eliminate irrelevant or noisy evidence (including formulas and embeddings) which may be introduced by meaningless entity pairs, e.g., Peach Tree, Pollination irrelevant to the original question.

We conduct experiments on AI2 Science Dataset to examine whether our inference method can acquire extra long-distance knowledge and bring improvement for read-world QA task. Moreover, in order to explore more deeply the effect of inference and focus on the questions that definitely need inference, we propose a new dataset, named as InfQAD. This dataset totally contains more than 11,000 real-world examination questions in seven subjects with two languages (English and Chinese), where questions that can be answered only by simple retrieval have been filtered out. The experimental results on InfQAD show that logic inference and embedding-based method concentrate on different aspects of questions and they can complement each other.

In summary, the contributions of this paper are shown as follows:

  We decouple KB-based inference from question answering by creating a virtual hypothesis and apply inference to answer complex questions, which not only utilizes long-distance semantic associations but also bridges the gap between natural language questions and hypotheses for inference.
  We create a new dataset from real-world examination questions to specifically evaluate the performance of inference methods on those complicated questions which need inference to resolve. It contains seven subsets of different subjects and may promote study on domain specific inference.
   We conduct an experiment on AI2 Science Questions Dataset to prove that inference can improve the performance of retrieval. After that, we compare several inference methods on InfQAD, and our method outperforms baselines.

## 2. KB-Based Inference

An inference task should contain a hypothesis and evidence, and inference is a process of collecting evidence to prove the hypothesis. For simple inference task on the KB, the hypothesis usually has the form of *r* (*h, t*), and the evidence can be a path, loop, or subgraph. There are mainly two types of models: probabilistic logic inference and knowledge graph embedding.

### 2.1. Probabilistic Logic Inference

Probabilistic logic inference utilizes various logic formulas to perform probabilistic inference. A logic formula has a head and a body, respectively, corresponding to hypothesis and evidence, noted as Body ⟹ Head. For example, Has (*x*, *y*) ∧ Feed (*y*, *z*) ⟹ Attract (*x*, *z*) is a formula which can infer Attract (Flower, Bee), and its head is Attract (*x*, *z*), and its body is an abstraction of evidence, Has (*x*, *y*) ∧ Feed (*y*, *z*).

Logic formulas usually are mined automatically from KB with confidence or weights. For a specific head or class of heads, bodies of formula are frequent structures on the KB, and mining such frequent structures is an important component in several probabilistic logic models, e.g., Markov Logic Network (MLN) [4]. Random walk algorithm is proposed to perform sampling frequent structures on knowledge graph, and the frequent structures are conceptualized as formula bodies. After that, the counts of formulas are used to calculate formulas' confidence or learn formulas' weights.  Algorithm 1 shows a general process of mining formulas on KB by random walk.

The algorithm takes a class of hypotheses *H* (*X*, *Y*) as input and starts random walks from the head entity *x* in each ground hypothesis (Lines 1–3). For example, the algorithm finds a path from Flower to Bee for Attract (Flower, Bee), e.g., $\text{Flower}\overset{\text{Has}}{⟶}\text{Pollen}\overset{\text{Feed}}{⟶}\text{Bee}$, and then the path is conceptualized as the body of formula (Lines 4-5), i.e., Has (*x*, *y*) ∧ Feed (*y*, *z*). After obtaining weighted formulas (Lines 6-7), probabilistic inference model employs the formulas as features and estimates the probability of the hypothesis as

(1) $$\begin{array}{c} p\left(h\right)=\frac{\phi \left(\sum_{f_{i}\in F}w_{i}x_{i}\right)}{\sum_{x_{i}\in X_{i}}\phi \left(\sum_{f_{i}\in F}w_{i}x_{i}\right)}, \end{array}$$

where *x*_*i*_ is a value about *f*_*i*_, e.g., truth value or count, and the denominator is a normalizing constant. *φ* is a nonlinear function; e.g., *ϕ* is an exponential function in Markov Logic Network.

### 2.2. Knowledge Graph Embedding

Knowledge graph embedding (KGE) model represents entities and relations as low-dimension numeric vectors or tensors and expects that arithmetical operations among embeddings can capture implicit relationships among elements. KGE can apply to inferring the hypothesis by defining a score function, noted as *F*_*r*(*h*, *t*)_; e.g., TransE [5] defines its score function as

(2) $$\begin{array}{c} F_{r\left(h,t\right)}=-\left\|E_{h}+E_{r}-E_{t}\right\|, \end{array}$$

where *E*_*h*_, *E*_*t*_, *E*_*r*_ are the embeddings of two entities and one relation, respectively. At training, the score of the triplet in KB is expected to be larger than triplets not in KB. After multiple rounds, embeddings are considered to contain implicit semantics and can perform inference.

## 3. Decoupling Inference from QA

The above KB-based inference methods all take a triplet *r* (*h*, *t*) in the KB as a hypothesis for inference, so if a complex question can be transformed into a triplet, KB-based inference method can solve it. This section describes a method that can distill a high-level triplet from the question as the hypothesis, and we call it virtual hypothesis.

### 3.1. Virtual Hypothesis

We try to transform a pair of question and option into a high-level triplet *R*_*q*_(*H*, *T*) and we propose the first assumption here.

**Assumption 1 .**

The virtual hypothesis *R*_*q*_(*H*, *T*) for a pair of question and option is the combination of all possible triplets *r* (*h*, *t*), where *r* is an implicit relation between an entity *h* in question and an entity *t* in option. Thus, *h *∈* H*, *t *∈* T*, and *R*_*q*_ is an integration of *r*.

We employ TransE model to explain the correction of Assumption 1. According to TransE, if *r* (*h*, *t*) is true, *h*+*r* ≈ *t*, and the distance of *r* (*h, t*) in distributed space, *D*_*r*(*h*, *t*)_=‖*E*_*h*_+*E*_*r*_ − *E*_*t*_‖, should be close to zero. We represent the sum of distances for all possible triplets as *D*_*q*_, e.g., triplets in Figure 1(b), and *D*_*q*_=∑_*i*=1_^*n*^‖*E*_*hi*_+*E*_*ri*_ − *E*_*ti*_‖. According to Triangle Inequality, ‖∑_*i*=1_^*n*^*E*_*hi*_+∑_*i*=1_^*n*^*E*_*ri*_ − ∑_*i*=1_^*n*^*E*_*ti*_‖ ≤ *D*_*q*_. However, *r*_*i*_ may have no clear definition; e.g., the relation between Flower and Pollination in Figure 1(c) is unknown. To handle this, we create a virtual relation type *R*_*q*_ and set *E*_*R*_*q*__=∑_*i*=1_^*n*^*E*_*r*_*i*__, so the distance of *R*_*q*_(*H*, *T*) is *D*_*R*_*q*__(*H*, *T*)=‖*EH*+*E*_*R*_*q*__ − *ET*‖, and *D*_*R*_*q*__(*H*, *T*) ≤ *D*_*q*_ which means the virtual hypothesis *R*_*q*_(*H*, *T*) is true if and only if all atomic hypotheses *r*_*i*_(*h*_*i*_, *t*_*i*_) are true. Thus, we believe the virtual hypothesis covers all semantics in atomic hypotheses, but there is temporarily no association between the virtual hypothesis and the original question. We propose the second assumption as follows.

**Assumption 2 .**

In distributed space, the embedding of virtual relation *R*_*q*_ is close to the embedding of the original question without entities.

According to Assumption 1, *R*_*q*_ is a combination of implicit relations between question entities and option entities, which should be what the question describes. For example, a simple question “Who is first emperor of Tang Dynasty” can be represented by a triplet FirstEmperor (Tang Dynasty, *X*), and then “Who is first emperor of” describes the semantics of the relation type FirstEmperor. Therefore, Assumption 2 is reasonable, and we get the concrete definition of a virtual hypothesis *R*_*q*_(*H*, *T*)—*H* and *T*, respectively, are the entity sets in question and option, and *R*_*q*_ is the question without entities. At present, we have distilled a high-level triplet from a pair of question and option as the simple hypothesis for inference.

### 3.2. Logic Inference with Virtual Hypothesis

When we employ logic formulas to infer a normal triplet *r* (*h*, *t*) on the KB, we select applicative logic formulas by the relation *r*; e.g., the formula Has (*x*, *y*) ∧ Feed (*y*, *z*) ⇒ Attract (*x*, *z*) which is obtained from the instance Has (Flower, Pollen) Feed (Pollen, Bee) ∧ Attract (Flower, Bee) can be also used to infer Attract (Honeycomb, Bear). However, the virtual relation *R*_*q*_ is specific to a question, and no two *R*_*q*_ can share their formulas. To capture associations between *R*_*q*_ (*H*, *T*) and formulas, we propose the third assumption.

**Assumption 3 .**

If a formula *f* can be used to infer *R*_*q*_(*H*, *T*), the body of *f* should be close to *R*_*q*_ in distributed space.

Intuitively, some formulas have such a property; e.g., in Father (*x*, *y*) ∧ Father (*y*, *z*) ⇒ Grandfather (*x*, *z*), Father + Father should be close to Grandfather. More formally, we still employ TransE to explain the correction. For a formula *f*, *r*_1_(*x*_1_, *x*_2_)∧…∧*r*_*n*_(*x*_*n*_, *x*_*n*+1_)⇒*r*_1_(*x*_1_, *x*_*n*+1_), if the body of f is true, and *r*_*i*_(*x*_*i*_, *x*_*i*+1_) is true. Thus, *E*_*x*_*i*__+*E*_*r*_*i*__=*E*_*x*_*i*+1__, and then *E*_*x*_*i*__+∑_*i*=1_^*n*^*E*_*r*_*i*__=*E*_*r*_*n*+1__. We define the embedding of *f* as *E*_*x*_*f*__=∑_*i*=1_^*n*^*E*_*r*_*i*__, so *E*_*x*_1__+*E*_*r*_*f*__=*E*_*x*_*n*+1__. Since the head of *f* is also true, *E*_*x*_1__+*E*_*r*_*r*__=*E*_*x*_*n*+1__, so we get *E*_*f*_=*E*_*r*_, which is exactly Assumption 3.

We search paths from any entity in *H* to any entity in *T* and transform them into the body of formulas *f*. Then, we represent *f* as *E*_*f*_=∑_*i*=1_^*n*^*E*_*r*_*i*__ and calculate the similarity between *f* and the virtual relation *R*_*q*_ as

(3) $$\begin{array}{c} \text{Sim}\left(f,R_{q}\right)=\left\|E_{f}-E_{R_{q}}\right\|. \end{array}$$

Finally, we employ the similarity Sim (*f*, *R*_*q*_) to replace the count of *f* 's instances between *H* and *T*, so equation (1) changes to

(4) $$\begin{array}{c} P_{l}\left(h\right)=\frac{1}{Z}\phi \left(\sum_{f_{i}\in F}w_{i}*-\left\|E_{h}-E_{f_{i}}\right\|\right), \end{array}$$

where *Z* is the normalizing constant.

### 3.3. KB Embedding with Virtual Hypothesis

To adapt KB embedding model, i.e., TransE, to the virtual hypothesis, we give the fourth assumption.

**Assumption 4 .**

In distributed space, the entity set *H* in question is close to the entity set *T* in option under the translation of virtual relation *R*_*q*_.

Assumption 4 can be deduced from Assumption 2. In Assumption 2, *E*_*R*_*q*__ is the embedding of the original question without entities, so *E*_*R*_*q*__+*E*_*H*_ is exactly the embedding of the question *E*_*q*_. *T* is the set of entities in option, so *E*_*T*_ is close to the embedding of the option *E*_*o*_. Thus, ‖*E*_*H*_+*E*_*R*_*q*__ − *E*_*T*_‖ ≈ ‖*E*_*q*_ − *E*_*o*_‖, and its right part means question is close to option in distributed space, which is a truth when the option is the correct answer. Therefore, TransE with virtual hypotheses is a kind of text inference.

### 3.4. Joint Objective Formalization

To utilize different types of evidence from both logic formulas and KB embedding, we build a joint objective *G* which combines *P*_*l*_ and *P*_*h*_ as

(5) $$\begin{array}{c} G\left(h\right)=\alpha P_{l}\left(h\right)+\beta P_{l}\left(n\right), \end{array}$$

where *α* and *β* are hyper-parameters, and *α* + *β* = 1. To simultaneously learn word embeddings and KB embeddings, we minimize a margin-based ranking criterion over the training set as

(6) $$\begin{array}{c} ℶ=\sum_{h^{\prime }\in O_{w}}{\left[G\left(h^{\prime }\right)+\gamma -G\left(h\right)\right]}_{+}, \end{array}$$

where *γ* is a margin, *O*_*w*_ is the wrong option set, and *h*′ is a hypothesis formed by the question and a wrong option. The optimization is carried out by stochastic gradient descent with the additional L2 regularization on parameters.

## 4. Experiments

To explore whether our method would acquire long-distance knowledge and bring an improvement for read-world QA task, we combine our methods with a retrieval-based method and conduct an experiment on AI2 Science Question Dataset1. After that, to further explore the effect of inference and focus on questions which need inference, we create an Inference Question Answering Dataset (InfQAD), in which questions cannot be answered by search or retrieval. After that, we compare several types of inference on InfQAD.

### 4.1. Evaluation on AI2 Science Questions

#### 4.1.1. Dataset

AI2 Science Question Dataset contains 5343 4-way multiple-choice science questions without diagrams at the elementary and middle school levels, and they are divided into Train, Dev, and Test.  Table 1 shows the statistics of this dataset. We employ three types of resources as backgrounds, including Wikipedia, Freebase [8], and ConceptNet [9].

#### 4.1.2. Setting

We implement a retrieval method based on Lucene which is an open-source information retrieval software library, and we employ the method to build reverse index on the whole Wikipedia dump. We concatenate a question with an option as the query for retrieval and calculate the average of Top-3 scores. We rank options by the average scores, and the highest one is the final answer. All questions and options are preprocessed by CoreNLP [10]. For our logic-based method, we use simple maximal matching algorithm to extract entities from the question and options, respectively. When collecting ground formulas for hypotheses, we employ a typical random walk algorithm to run on both Freebase and ConceptNet and limit the maximal length of formula to 4. For our embedding-based method, we represent a question by the sum of embeddings of its words which were pretrained by GloVe [11] with 100 dimensions. We combine results of the retrieval-based method and our methods by two steps:

1. For each solver, we normalize scores across the answer options for a given question
2. We send normalized scores into a classifier which can output correct/incorrect with confidence, and the correct option with the maximal confidence is treated as the final answer

#### 4.1.3. Results and Analysis

We show the accuracy of methods in Table 2, where +Emb and +Logic represent adding embedding and logic formulas, respectively. We can obtain the following observations:

1. Combining two types of inference methods with retrieval can improve performances, which proves that decoupling inference by virtual hypothesis is effective and KB-based inference can utilize a mass of extra long-distance knowledge to improve the performance of the retrieval method.
2. The promotion on middle school dataset is more obvious than that on elementary dataset, which implies middle school examination is more difficult than elementary examination, and difficult questions need inference more.
3. Only adding embedding into retrieval leads to performance reduction. We think the reason is that wrong answers from the unsuitable solver may affect others. Logic inference tends to refuse to answer with low confidence, while embedding method gives answers in any case, which may distract retrieval from giving the correct answer.

### 4.2. Inference QA Dataset Construction

Retrieval-based method achieves a good performance on AI2 dataset, but the experiment above shows that retrieval may affect the further exploration of inference. Therefore, we propose constructing a new Inference Question Answering Dataset, named as InfQAD, which only contains complicated questions that need inference. InfQAD contains 11,393 examination questions in seven subjects with two languages (five subjects in English and two subjects in Chinese), and in the dataset, questions that only need retrieval have been filtered out.  Table 3 shows the statistics of InfQAD. We construct InfQAD by two major steps: question collection and question filtration.

For five subjects in English, we download questions from the CK12 website2. There is a downloadable quiz in almost every topic, and the quiz usually contains ten questions. We only keep 4-way multiple-choice questions without diagrams as AI2 does. For two subjects in Chinese, we collect about 200 senior high school entrance examination papers, which also only keeps 4-way multiple-choice questions without diagrams.

To filter out questions which can be answered by retrieval, we treat Lucene as a standard retrieval method and employ it to score each pair of question and option. We sort questions according to the difference between the score of its correct answer and the maximal score of its incorrect option in descending order. We remove top questions and make the accuracy of Lucene on the rest of questions 25% which equals the accuracy of random choice. We believe Lucene fails for the rest of the questions, and they can be viewed as questions that need inference to resolve, approximatively.

### 4.3. Evaluation on Inference Questions

#### 4.3.1. Methods Compared

We compare different kinds of methods on InfQAD, including probabilistic logic inference (in Table 4(b)), embedding-based inference (in Table 4(c)), and ensemble inference (in Table 4(d)). Probabilistic logic inference includes the following:

1. Traditional MLN [4], which treats all hypotheses as the same relation, and questions share all weighted formulas
2. Cluster-based MLN, which first clusters questions by the similarities between questions and then trains an MLN Model for each cluster of questions
3. Our method described in Section 3.2, noted as VHLogic

Embedding-based inference contains two approaches which both estimate the similarity between questions and options but employ different methods of representing text, i.e., SUM [12] and GRU [13]. Ensemble inference is combining VHLogic with two methods of embedding-based inference as in Section 3.4. We also add the results of random choice (Random) and the retrieval-based method (Retrieval) into the result in Table 4(a) for comparison.

#### 4.3.2. Setting

We implement MLN as described in [14]. We implement SUM method and employ a GRU tool in Java3. We still use pretrained word vectors by GloVe [11] with 100 dimensions for English questions and train word vectors with 100 dimensions on Baidu Baike for Chinese questions. In this experiment, we only employ ConceptNet as the KB for both English and Chinese questions.

#### 4.3.3. Results

We show the accuracy of methods in Tables 4 and 5, and we can obtain the following observations:

1. Comparing VHLogic with other logic inference methods in Tables 4(b) and 5(b), VHLogic has the best performance on almost all subsets, which indicates decoupling logic inference is effective, and the distributed similarity between hypotheses and formulas improves the performance of inference.
2. Comparing logic inference methods in Table 4(b) with SUM and GRU in Table 4(c), there is no obvious evidence that some kind of method could achieve better performance than another kind. It implies that different types of inference are better at questions in some subjects and may complement each other. The experimental results are also applicable to methods in Table 5(b) compared with Table 5(c).
3. Comparing ensemble inference methods in Table 4(d) with single methods in Tables 4(b) and 4(c), ensemble methods outperform single methods on almost all subsets, which proves that different types of inference concentrate on different aspects of questions and they can complement each other. The experimental results are also applicable to methods in Table 5(d) compared with Tables 5(b) and 5(c).

#### 4.3.4. Data Analysis

**Figure 2:**
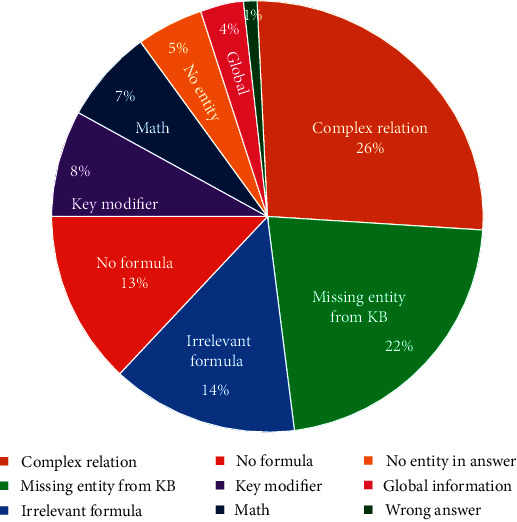
Statistics of breakdowns.

To analyze various causes of breakdowns, we sample 100 questions answered incorrectly by VHLogic and roughly classify them into several categories (shown in Figure 2):

1. *Complex Relation.* This category is that there is a relationship among more than two entities in the question, and the relationship is the key to answer the question. This category is the largest category, and 26% of questions belong to it.
2. *Missing Entity from KB*. This category is that there is a key entity missing from the KB, which leads to key formulas not being found. This category contains 22% of questions.
3. *No Entity in Answer*. This category is also about entities and contains 5% of questions. Answers of these questions contain no entity but numbers, modifications, or other elements.
4. *No Formula*. Sometimes, there are still no paths or useful formulas between entities, though entities in the question and the answer are both linked in KB. 13% of questions belong to this category.
5. *Irrelevant Formulas*. Irrelevant formulas are noise in inference process, which may disturb useful formulas, and this category has 14% of questions.
6. *Key Modifier*. Comparative degree, superlative degree, and other modifiers may be the key of answering the question, but these modifiers cannot be captured by VHLogic. There are 8% of questions belonging to this category.

There are other categories: 7% of questions belong to Math category which needs mathematical operations. 4% of questions need Global Information; e.g., the answer is All of the above, and there are 1% of questions with Wrong Answer.

## 5. Related Work

Our work is related to two types of work: question answering and KB-based inference. In recent years, various QA tasks and datasets have been emerging in an endless stream. WebQuestions [7], BAbI [15], and SimpleQuestions [16] mainly evaluate question understanding and assume the correct parsing results must be able to get the correct answer. MCTest [17] contains questions with 4 answer choices per question like ours, but each question and its answer in MCTest come from a given story. The Children Book Test [18] and CNN/Daily Mail dataset [19] view cloze test as a kind of QA task, while SQuAD [20] also employs word or phrase in original text as the answer. AI2 Science Dataset [1] is the most related to our InfQAD, but InfQAD only contains the questions which need inference. Aristo [21] is a QA system for science questions which combines 5 solvers including IR, MLN [22], and other inference methods. Aristo extracts inference rules from texts by patterns, while our method mines formulas from KB.

On the other hand, knowledge base (KB) organizes information as a graph. Graph learning has been widely used in many other fields such as image classification [23, 24]. KB-based inference mainly has two types of approaches: probabilistic logic inference and knowledge graph embedding. Besides MLN [1] mentioned in the previous sections, Inductive Logic Programming (ILP) [3], PSL [13], and PRA [12] all belong to probabilistic logic inference models. These models obtain logic formulas from knowledge graph and perform probabilistic inference, but they cannot handle virtual hypothesis as VHLogic does. TransE, RESCAL [6], TransH [25], and TransR [26] are all embedding-based methods, and relative to our method, they employ different similarity functions to calculate scores of hypotheses. There are also several methods to represent formula by embeddings, including PTransE [27], RNN [28], and ProPPR + MF [29–31], while these methods only represent formulas, but do not simultaneously represent texts as our method does.

## 6. Conclusion

We propose a method to decouple KB-based inference from real-world QA by creating a high-level triplet on the KB named as virtual hypothesis, and adjust logic-based and embedding-based method to inferring it. The experimental results prove that our method is effective and is a promising method to apply inference to QA. In addition, we propose a specialized question answering dataset only for inference, named as InfQAD. We compare various inference methods on InfQAD and find that different types of inference are skillful in different subjects and combing them will improve the performance. At last, we analyze various causes of breakdowns, which can be helpful for the future study on domain specific inference. In the future, there are two aspects of our work that need deeper exploration. We try to find a better way to represent virtual hypotheses and try to reconstruct textual knowledge base to better capture long-distance evidence as formulas.

## Figures and Tables

**Algorithm 1:**
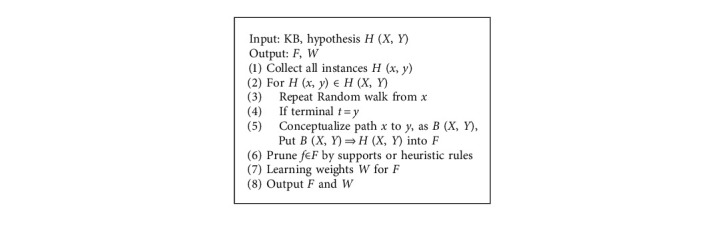
Mining formulas by random walk.

**Table 1:** Statistics of AI2 Science questions.

Dataset	Train	Dev	Test	Total
Elementary	574	143	717	1636
Middle	1583	485	1639	3707

**Table 2:** Accuracy of methods on AI2 Science questions.

Methods	Elementary (%)	Middle (%)
Retrieval	45.33	40.02
+Emb	43.65	39.29
+Logic	45.33	40.81
+Emb + Logic	45.46	41.12

**Table 3:** Statistics of InfQAD.

Datasets	Train	Dev	Test	Total
*English*				
Biology	242	48	193	483
Chemistry	749	149	599	1497
Earth	737	221	516	1474
Life	421	126	295	842
Physical	293	87	205	585

*Chinese*				
Biology	1919	300	1618	3837
History	1338	300	1037	2675

**Table 4:** Accuracy of methods on InfQAD Chinese.

	Datasets	Chinese	
	Methods	Biology (%)	History (%)
4(a)	Random	25.00	25.00
	Retrieval	24.43	27.17

4(b)	MLN	32.14	29.60
	MLN (cluster)	28.18	28.45
	VHLogic	35.04	35.10

4(c)	SUM	44.44	42.62
	GRU	40.30	36.84

4(d)	VHLogic + SUM	44.31	42.81
	VHLogic + GRU	40.60	36.07

**Table 5:** Accuracy of methods on InfQAD English.

	Datasets	English				
	Methods	Biology (%)	Chemistry (%)	Earth (%)	Life (%)	Physical (%)
5(a)	Random	25.00	25.00	25.00	25.00	25.00
	Retrieval	25.91	23.04	24.03	27.46	23.90

5(b)	MLN	27.46	23.87	27.71	24.07	31.71
	MLN (cluster)	**32.64**	23.54	27.32	28.14	26.83
	VHLogic	31.61	25.21	**34.11**	**31.19**	34.15

5(c)	SUM	29.53	26.38	33.53	24.75	37.07
	GRU	29.53	**32.22**	32.55	30.17	**39.02**

5(d)	VHLogic + SUM	34.20	27.88	**35.85**	30.85	37.56
	VHLogic + GRU	**34.72**	**33.39**	34.10	**31.53**	**39.51**

## Data Availability

The dataset concerns commercial confidentiality, so it is not suitable for publishing.
